# Electrochemical Oxidation of Methyl Orange in an Active Carbon Packed Electrode Reactor (ACPER): Degradation Performance and Kinetic Simulation

**DOI:** 10.3390/ijerph19084775

**Published:** 2022-04-14

**Authors:** Jing Hou, Xue Li, Yuting Yan, Lizhang Wang

**Affiliations:** Environmental Energy Engineering (*E3*) Workgroup, School of Environment Science and Spatial Informatics, China University of Mining and Technology, Xuzhou 221116, China; houjing1207@126.com (J.H.); xueli_cumt@126.com (X.L.); yanyt36999@163.com (Y.Y.)

**Keywords:** methyl orange, electrocatalytic oxidation, ACPER, anode expansion coefficient, phase-reaction kinetics model

## Abstract

The efficient removal and kinetic modelling of methyl orange (MO) degradation using an electrocatalytic oxidation method in an activated carbon (AC) packed electrode reactor (ACPER) were conducted. A significantly high (81.2%) chemical oxygen demand (COD) and 100.0% MO decolorization efficiency were observed under the experimental conditions of current density of 3.0 mA·cm^−2^, flow velocity of 0.3 L·h^−1^, and treatment duration of 1.68 h using a *β*-PbO_2_/Ti anode. The high removal efficiency is ascribed to the anode expansion effect after AC packing. The anode expansion coefficient (*λ*) of the ACPER was calculated to be 0.63 from the cyclic voltammetry (CV) measurement, which means the further current utilization for MO oxidation. Based on the current utilization efficiency on anodic and particle electrode surfaces, a phase-reaction kinetics model was proposed for the simulation of MO COD removal efficiency. Our simulation results showed that the newly established average current efficiency (*ACE*) and energy consumption (*E_sp_*) model well matched the MO experimental degradation data. Our work broadens the scope of the application of ACPER in the treatment industry wastewater containing organics and provides a new strategy for the energy utilization evaluation during the removal of organic matter by electrocatalytic oxidation.

## 1. Introduction

The past few decades have witnessed widespread and rapid global development of textile industries, and thus a large amount of dye containing effluent is discharged. Due to its high organic matter concentration, large molecules, and poor biodegradability, dye containing effluent is recognized as one of most persistent and difficult to treat types of wastewater [1,2,3,4,5]. The purification of dye containing effluent poses serious challenges for aquatic ecosystem sustainability and public health [6]. Several technologies have been pursued for the elimination of organic dyes from polluted water, including resin adsorption [7], membrane separation [8], and wet catalytic oxidation [9]. However, due to their low removal efficiency, high treatment cost, and the production of secondary pollutants, considerable work remains to be done. The electrocatalytic oxidation technique is a multifunctional advanced oxidation processes (AOPs) which has been widely used in organic matter degradation treatments, for example in the treatment of phenol, antibiotic, and perfluorooctanoic acid containing wastewater [10,11,12]. The generated hydroxyl radicals (·OH) can realize the mineralization or decomposition of organic matter [13]. Baddouh et al. [14] and Li et al. [15] also documented the degradation feasibility of dye containing wastewater with the electrocatalytic oxidation method. Nevertheless, higher removal efficiency, improved space-time yield, and superior energy efficiency are still the prerequisites for a practical application.

An activated carbon (AC) packed electrode reactor (ACPER) that features an increased reaction interface and enhanced mass transfer rate can significantly improve the organic matter degradation efficiency compared with instant electrocatalytic oxidation [16,17]. Latifi et al. [18], Wang et al. [19], and Ghezel et al. [20] have discovered the much higher COD removal efficiency in the treatment of wastewater containing methylene blue, citric acid, and acid red 18. Moreover, particle electrodes form countless tiny cells and participate in the degradation of pollutants [21]. This leads to changes in charge transfer and mass transfer, which can affect the oxidation current utilization ratio. Conventional kinetic strategies for electrochemical oxidation include pseudo first-order kinetics and second-order kinetics [22,23]. These are widely used but have some limitations, such as the failure to consider the oxidation current utilization ratio. Therefore, a phase-reaction kinetics model based on the current utilization efficiency on the anode and particle electrode surfaces is proposed based our previous work [24]. To date, we have carried out the model simulation with phenol, landfill leachate and wastewater containing pyridine-derivatives, etc. [24,25]. However, to our knowledge, there is little research on the phase-reaction kinetics model of azo dye wastewater. Methyl orange (MO) is a typical azo dye, which is formed by diazotizing p-aminobenzene sulfonic acid and coupling with *N*,*N*-dimethylaniline. It is widely used in the production of printed and dyed textiles, and its toxicity, carcinogenic and teratogenic effects on the human body are still a serious problem. The efficient removal of MO from wastewater is, therefore, an urgent task.

In this work, we attempted to improve the MO degradation efficiency through the ACPER using a *β*-PbO_2_/Ti anode. Then, a suitable kinetic model was adopted for the simulation of the experimental results, including the average current efficiency (*ACE*) and energy consumption (*E_sp_*). The results indicate that this technology is a feasible method to reduce the COD values of wastewater. The experiment data are consistent with the model calculation outcome. The results in this study may contribute to the application of ACPER and enrich the content of kinetic model research.

## 2. Materials and Methods

### 2.1. Reagents and Materials

Pure titanium sheet (99.9%, length: 100 mm and width: 100 mm), platinum plate (99.9%, length: 40 mm and width: 40 mm), and *β*-PbO_2_/Ti plate (length: 100 mm and width: 100 mm; length: 20 mm and width: 20 mm) were purchased from Baoji Qixin Titanium Industry Co., Ltd. (Xi’an, China). Methyl orange (MO), Na_2_SO_4_ were analytical grade, purchased from Tianjin Fuchen Chemical Reagent Co., Ltd. (Tianjin, China). All chemicals applied in the experiments were used as received without any further purification. For solution preparation and data measurement, deionized water was used.

### 2.2. Electrochemical Properties Test

The standard three-electrode system of the CS310 electrochemical workstation was used for electrochemical performance testing. The test working electrode was a *β*-PbO_2_/Ti plate with a length and width both of 20 mm; the counter electrode was a platinum sheet; the reference electrode was a saturated calomel electrode (SCE), and the filler particles were activated carbon (AC) with a diameter of 3 mm to 5 mm. Cyclic voltammetry (CV) and Electrochemical impedance spectroscopy (EIS) tests were performed on this system in a 3% Na_2_SO_4_ solution containing 450 mg·L^−1^ MO. The scanning speed of the system CV curves was 5 mV·s^−1^, 10 mV·s^−1^, 20 mV·s^−1^, 50 mV·s^−1^ and 100 mV·s^−1^; the scanning potential range was 0~2.0 V vs. SCE; the polarization test scanning speed was 5 mV·s^−1^, potential range 0~2.0 V vs. SCE; the EIS test potential was bias 1.80 V vs. SCE higher than the oxygen evolution potential (Figure S1). In order for the oxidation reaction and oxygen evolution reaction to occur in the system. The frequency range was 100 kHz~0.01 Hz. All the tests were performed at room temperature.

### 2.3. Bulk MO Simulated Wastewater Degradation Experiment

**Figure 1:**
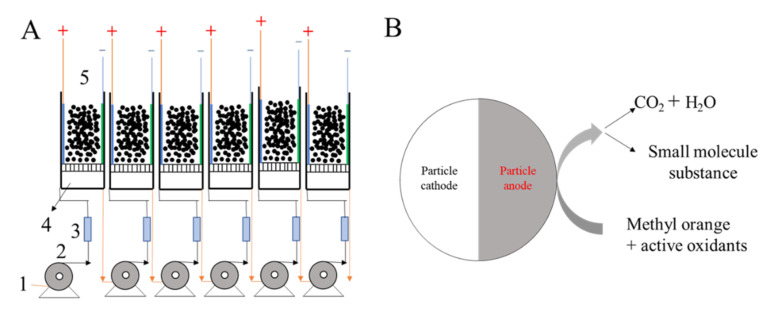
Schematic of the experimental setup (**A**) and particle electrode structure (**B**). (1) Influent; (2) metering pump; (3) flowmeter; (4) water reservoir; (5) reaction zone.

The electrochemical oxidation apparatus consisted of a DC power supply, a peristaltic pump, and the ACPER. The dimensions (length × width × height) of the electrocatalytic reactor were 100 mm × 100 mm × 50 mm, and the AC filling volume was 415 mL. A *β*-PbO_2_/Ti electrode was used as an anode and a titanium sheet plate was used as cathode. A *β*-PbO_2_/Ti electrode was used because its conductivity is higher than that of an *α*-PbO_2_/Ti electrode, and it is more widely used in the field of electrocatalytic oxidation. The composition of the inlet water solution was 450 mg·L^−1^ MO and 3% Na_2_SO_4_, which was selected as the supporting electrolyte in this study. The inlet water flow rate *Q* was set to 0.3 L·h^−1^, 0.5 L·h^−1^, and 1.0 L·h^−1^ and the current density *J* was 3.0 mA·cm^−2^, 5.0 mA·cm^−2^, and 10.0 mA·cm^−2^. The wastewater was discharged after six treatments, and each stage was sampled for measurement. The reactor and AC configuration are shown in Figure 1.

### 2.4. Analytical Method

The calculation method of the parameters used in the kinetic model is based on previous literature [26,27]. The foundation of the kinetic theory is that the input electric energy can be effectively used by the organic matter oxidation process and reduce the oxidation-reduction potential of the system. It is more appropriate to measure this process by the COD. The total organic carbon (TOC) index reflects the change level of the system organic carbon. Even if the COD concentration in the wastewater is low, the TOC value may still be high. Therefore, it is suitable to use COD as an indicator, and it is tested by the standard dichromate methods (Supplementary Materials). A UV-visible spectrophotometer (Shimadzu UV-1700, Shanghai, China) was used to obtain the absorbance spectra of MO solutions. The decolorization rate of MO was monitored by measuring the decrease of absorbance at the maximum visible wavelength (~464 nm). The calculation method of MO concentration and decolorization rate are shown in the Supplementary Information (SI) (Figures S1 and S2).

## 3. Results and Discussion

### 3.1. Degradation Performance Evaluation of Bulk MO Simulated Wastewater

**Figure 2:**
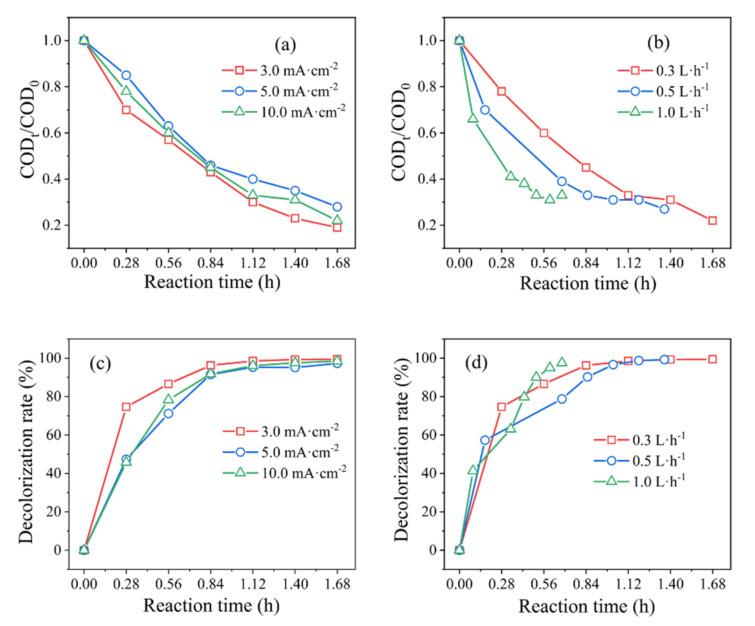
Effects of (**a**,**c**) current density and (**b**,**d**) flow rate on *COD_t_/COD*_0_ values and decolorization efficiency on the *β*-PbO_2_/Ti anodes. Experimental conditions: 450 mg·L^−1^ MO + 3% Na_2_SO_4_, (**a**,**c**) flow rate of 0.3 L·h^−1^, (**b**,**d**) current density of 10.0 mA·cm^−2^, and room temperature.

Figure 2a,c depict that the *COD_t_/COD*_0_ values and decolorization efficiency slightly increased with the strengthening of current density from 5.0 to 10.0 mA·cm^−2^. Effective MO removal (81.15%) and decolorization (100%) were obtained at the lowest current (3.0 mA·cm^−2^) after 1.68 h of electrolysis. This is because, although high current density can generate more active oxidants for oxidizing organics, it leads to an increased side reaction of oxygen evolution [28]. The influence of flow rate upon the electrochemical oxidation and decolorization reaction are shown in Figure 2b,d from 0.3 L·h^−1^ to 1.0 L·h^−1^. The results show that the *COD_t_/COD*_0_ values and decolorization efficiency decreased with the reduction in flow rate. Obviously, residence time increased as the flow rate was reduced, which is conducive to organic matter reactions [29].

The oxidative degradation of MO simulated wastewater was mainly attributed to the indirect oxidation of the anode (Equations (1)–(3)) as well as the direct electro-oxidation on the polarized AC and indirect electro-oxidation with in situ generation of strong oxidants (e.g., H_2_O_2_) (Equation (4)). The reaction formula is as follows [30,31]:

(1) $$N+H_{2}O\to N(\cdot \mathrm{OH})+H^{+}+e^{-}$$

(2) $$N(\cdot \mathrm{OH})\to \mathrm{NO}+H^{+}+e^{-}$$

(3) NO+R → N+RO

(4) $$O_{2}+H^{+}+2e^{-}\to H_{2}O_{2}$$

where N is the anode; N(·OH) is the higher oxide; and R is the pollutant.

The space-time yield (*Y_st_*), which represents the degradation amount of MO per unit volume and per unit time by using ACPER, was calculated by Equation (5) [16].

(5) $$Y_{\mathrm{st}}=\frac{\mathrm{dm}}{\mathrm{dt}\cdot \mathrm{dV}}$$

**Figure 3:**
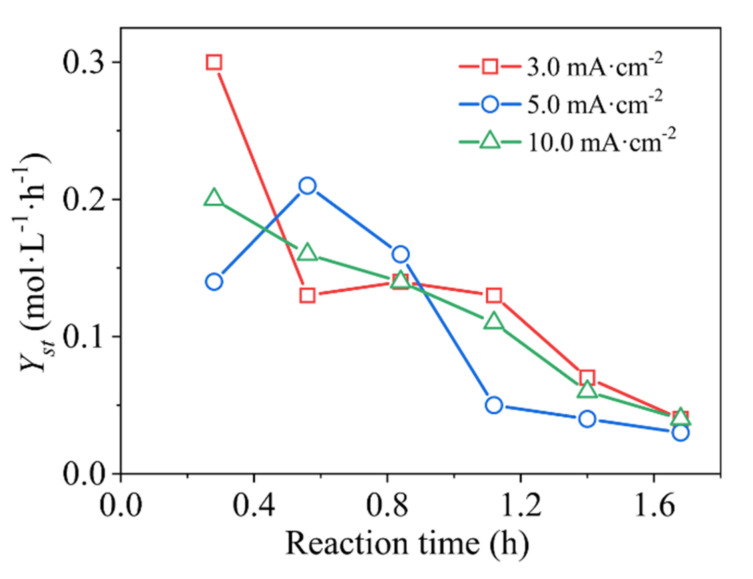
The reaction time dependence of the *Y_st_* under different current densities on the *β*-PbO_2_/Ti anodes. Experimental conditions: 450 mg·L^−1^ MO + 3% Na_2_SO_4_, flow rate of 0.3 L·h^−1^, and room temperature.

Studies have shown that the *Y_st_* of ACPER is at least 100 times higher than that of two-dimensional electrode reactors [32]. Figure 3 depicts the relationship between *Y_st_* and *t* at different current densities. It can be seen that the highest *Y_st_* value (0.3 mol·L^−1^·h^−1^) appears at a low current density of 3.0 mA·cm^−2^, which is consistent with the results of *COD_t_/COD*_0_ and decolorization efficiency. Although there are cases in which *Y_st_* is lower at this current density, the overall value is higher than other current densities. At the beginning of the electrocatalytic oxidation, high concentration of MO can effectively reach the anode surface from the bulk solution and fully react with the active oxidants generated by the anode. As the reaction progresses, the *Y_st_* decreases rapidly. The reduced concentration of MO means that mass transfer is restricted, and the oxygen evolution side reaction has occurred. Exceptionally, due to the use of AC particle electrodes, the *Y_st_* is slightly increased during the degradation process of MO at 3.0 mA·cm^−2^ and 5.0 mA·cm^−2^ compared with a traditional two-dimensional electrochemical reactor.

**Figure 4:**
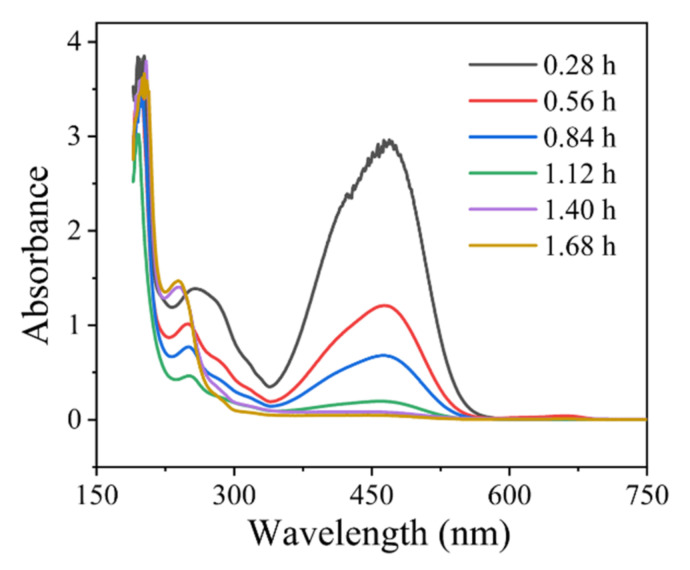
UV-Visible spectrum with different residence times of MO. Experimental conditions: 450 mg·L^−1^ MO + 3% Na_2_SO_4_, flow rate of 0.3 L·h^−1^, current density of 10.0 mA·cm^−2^, and room temperature.

The UV-Vis spectrum of the MO solution with different dwelling time exhibited two bands: one in the visible region (~464 nm) is associated with the azo structure that makes MO develop color, and another band (~270 nm) is associated with benzene ring structure (Figure 4) [33]. With the progress of degradation, the absorption peak of ultraviolet and visible light at 464 nm rapidly decreased. The absorption peak at 270 nm of MO dropped less because it was difficult to degrade the benzene ring structure. After 1.40 h, the azo structure was destroyed, and the molecules were also decomposed. This result is consistent with the observation that the solution becomes transparent during the experiment.

### 3.2. Electrochemical Properties Test of Anode and AC Particle Electrode

**Figure 5:**
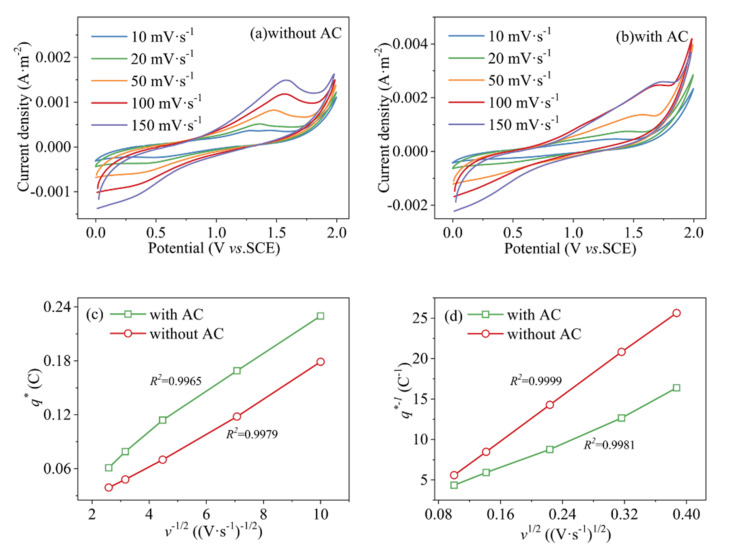
CV curves of (**a**) without and (**b**) with AC for *β*-PbO_2_/Ti anode with different sweep speeds in 450 mg·L^−1^ MO and 3% Na_2_SO_4_ solution, and the corresponding relationships between q^*^(v) and *v* (**c**,**d**).

In addition to the aforementioned results, electrochemical degradation performance of ACPER was also affected by the particle electrode and anode materials. Figure 5a,b display the CV curves of the solution at different sweep speeds. The time scale of the CV tests is controlled by the change of the potential scan rate. As the scan rate increases, the time scale of the CV test decreases and the peak current increases [34]. It can be seen from Figure S1 that the oxygen evolution potential (OEP) of the *β*-PbO_2_/Ti electrode is 1.70 V vs. SCE. Compared with other metal electrodes studied [30,35,36], the larger OEP electrode is not apt to adsorb oxidizing agents and has strong catalytic activity [37]. The integrated area of these curves represent the electrode active surface area and it increases after adding AC particles, which is conducive to the contact of active oxidants and MO [38]. Additionally, the number of active sites on the electrode surface is determined by the total volt-ampere energy (q^*^_t_), q^*^_t_ is composed of the inert voltampere energy q^*^_i_ and the active volt-ampere energy q^*^_o_ [38]. Their relationship can be expressed by Equations (6)–(8) [38,39]:

(6) $$q_{t}^{*}=q_{i}^{*}+q_{o}^{*}$$

(7) $$q^{*}=q_{o}^{*}+k_{1}\cdot v^{-1/2}$$

(8) $$q^{*-1}=q_{t}^{*-1}+k_{2}\cdot v^{1/2}$$

where *v* is the scan rate, V·s^−1^; q^*^(v) is the charge of the integrated area of the CV curve, C; and *k*_1_, *k*_2_ are constants.

According to Figure 5c,d combined with Equations (3) and (4), it can be determined that the active voltammetric powers q^*^_o_ are 28.5 mC·cm^−2^ and 17.5 mC·cm^−2^ with and without AC, respectively. In comparison, the former being 1.63 times the latter. Therefore, excluding the background feed current obtained through the anode, the anode expansion coefficient *λ* (area ratio of particle electrode to metal electrode under filler conditions) is 0.63. This value can quantitatively describe the ability of AC particles to expand the electrode area.

**Figure 6:**
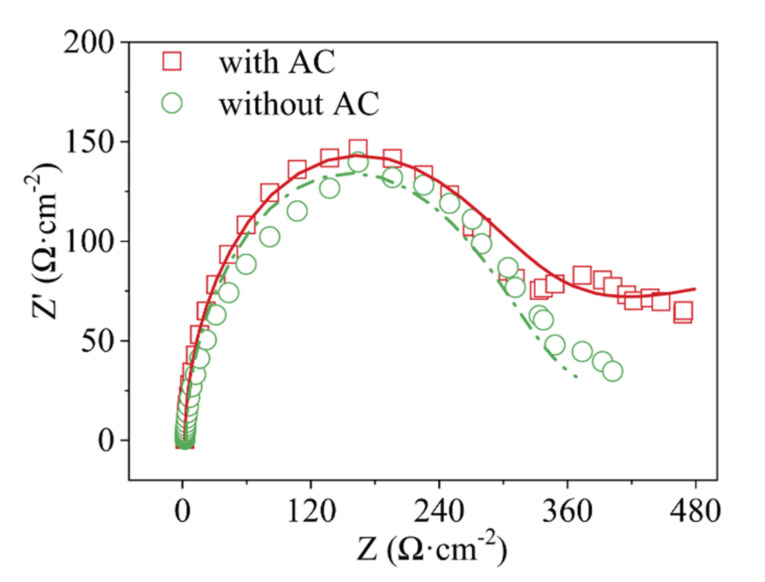
*β*-PbO_2_/Ti anodized methyl orange EIS and Nyquist fit (Bias 1.80 V vs. SCE; counter electrode platinum plate (length × width = 40 mm × 40 mm); reference electrode SCE; MO concentration 450 mg·L^−1^, 3% Na_2_SO_4_. Points and lines represent experimental and fitted values, respectively).

ZsimpWin software (3.30d, Bruno Yeum, ph.D., Ann Arbor, MI, USA) was used to fit the EIS spectrum of *β*-PbO_2_/Ti electrode with/without AC in 3% Na_2_SO_4_ + 450 mg·L^−1^ MO simulated solution. The equivalent circuit of the MO electrocatalytic oxidation process can be described by *R_s_(C(W(R_oe_(R_ct_Q))))* circuit model [24]. Among them, *R_s_* represents the resistance of the solution, and *R_ct_* represents the electron transfer resistance on the electrode surface during the oxygen evolution process. *R_oe_* is organic oxidation resistance. The fitted graph is presented in Figure 6. The fitting values of each component are listed in Table 1. Compared with the system without AC, the *R_oe_* increases and the *R_ct_* decreases after adding AC. The reason for this phenomenon is the formation of tiny galvanic cells between ACs, which are conducive to the transfer of electrons, enhances the reaction current for organic degradation, and partially inhibits the oxygen evolution reaction.

In order to quantitatively express the current utilization efficiency on anodic and particle electrode surface, we introduced the parameters *γ* and *β*, which can characterize the selective oxidation coefficient of the anode and the effective current proportion of the particle electrode. The value of (*γ* + *β*) or *γ* reflected in Table 1 can be calculated by the relationship between *R_oe_* and (*R_oe_* + *R_ct_*) (Equation (9)). To characterize the performance of the *β*-PbO_2_/Ti anode and AC particle electrode on MO degradation, the *γ* and *β* were 0.80 and 0.20, respectively.

(9) $$(\gamma +\beta )\mathrm{or}(\gamma )=\frac{R_{\mathrm{oe}}}{R_{\mathrm{oe}}+R_{\mathrm{ct}}}$$

### 3.3. Phase-Reaction Kinetics Model Simulation of Energy Efficiency

To judge whether the phase-reaction kinetic model, based on the current utilization efficiency on anodic and particle electrode surface, is suitable for describing the treatment of MO wastewater by ACPER, we calculated the energy efficiency of the degradation process, including the average current efficiency (*ACE*) and energy consumption (*E_sp_*).

**Figure 7:**
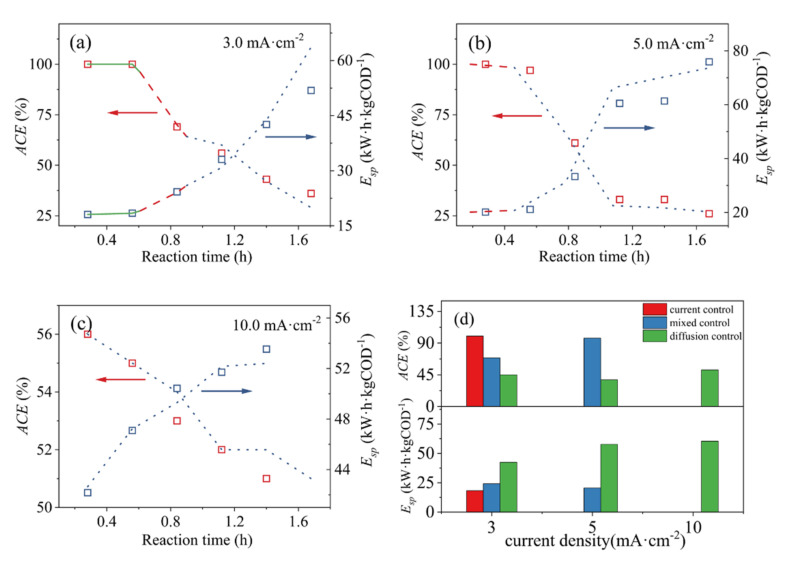
Dependence of the normalized *ACE* and *E_sp_* on reaction time against the applied current densities on the *β*-PbO_2_/Ti anode: (**a**) 3.0 mA·cm^−2^; (**b**) 5.0 mA·cm^−2^; (**c**) 10.0 mA·cm^−2^; (**d**) different stage. The solid, dashed and dotted curves represent the current control phase, mixed control phase and diffusion control phase, respectively, and the curves were obtained by model predictions by using the following variables: for 3.0 mA·cm^−2^, *α* = 0.45; for 5.0 mA·cm^−2^, *α* = 0.77; for 10.0 mA·cm^−2^, *α* = 1.56.

The current efficiency of organic matter oxidation has a decisive influence on energy consumption. Throughout the entire reaction process, an external power source provides energy to the system for the production of strong oxidants, movement, and oxidative degradation of organic matters, so the cost of the system is mainly power consumption [40]. The *ACE* and *E_sp_* are used to quantitatively describe the current utilization of the MO degradation process under different current densities, as shown in Figure 7.

According to the phase-reaction kinetic model, the process of the degradation for organic matter is divided into three stages: the current control stage, the current/diffusion mixed control stage and the diffusion control stage. The following model simulation results are analyzed according to the calculation formulas of each phase [24,25,38].

Current control phase: the oxidation of the anode and the particle electrode are both under current control, and the current components acting on the oxidation of the MO are fully utilized. The initial limiting current density (*i_lim_*_0_) can be expressed as *i_lim_*_0_ = *nFk_m_c*_0_ [27,41]. *ACE* can be described by Equation (10).

(10) $$\mathrm{ACE}=\frac{\int_{0}^{\tau }\mathrm{ICEdt}}{\tau }$$

In this phase, *ICE* = 1. *ICE* stands for instantaneous current efficiency, and *τ* represents the reaction time required to reach the target removal rate of organic matter. The reaction time required to reach the removal rate *R_E_* is Equation (11):

(11) $$\tau =\frac{R_{E}}{\alpha (\gamma +\beta )}\frac{{\varepsilon x}_{0}}{k_{m}}$$

where *n* is the number of electrons transferred (*n* = 4); *F* is the Faraday constant; *k_m_* is the mass transfer coefficient; *c*_0_ is the initial COD value of MO; *R_E_* is the MO removal rate; *ε* is the bed void fraction (*ε* = 0.17); *x*_0_ is the plate spacing (*x*_0_ = 5 cm); and *α* is the ratio of the input current to the initial limiting current density (*α* = *i/i_lim_*_0_).

Put Equation (7) into Equation (6) points to obtain *ACE* = 1.

The *E_sp_* calculation is obtained by using Equation (12):

(12) $$E_{\mathrm{sp}}=\frac{\mathrm{FU}}{8\times 3600(\gamma +\beta )}$$

where *U* is the voltage, and *F* has the same meaning as above.

Mixed control phase: the particle electrode organic oxidation is still under current control, while the anode organic oxidation is under mass transfer control. The *ICE* calculation formula at this stage is as follows:

(13) $$\mathrm{ICE}=\exp\left[-\frac{k_{m}}{{\varepsilon x}_{0}}t+\frac{\left(1-\alpha \gamma \right)}{\alpha (\gamma +\beta )}\right]$$

The reaction time of organic matter removal is calculated by Equation (15). Incorporate Equations (13) and (14) into Equation (10), and integrate the *ACE* formula. The *E_sp_* calculation is obtained by using Equation (16):

(14) $$\tau =\left[\frac{\left(1-\alpha \gamma \right)}{\alpha (\lambda +\beta )}-\ln\left(\frac{{1-R}_{E}+\alpha \beta }{\alpha (\lambda +\beta )}\right)\right]\cdot \frac{{\varepsilon x}_{0}}{k_{m}}$$

(15) $$\mathrm{ACE}=\frac{R_{E}}{1-\alpha \gamma \left[1+(1+\frac{\beta }{\gamma })\right]\ln\frac{{1-R}_{E}+\alpha \beta }{\alpha (\gamma +\beta )}}$$

(16) $$E_{\mathrm{sp}}=\frac{\mathrm{FU}}{8\times 3600(\gamma +\beta )}\frac{1-\alpha \gamma \left[1+(1+\frac{\beta }{\gamma })\right]\ln\frac{{1-R}_{E}+\alpha \beta }{\alpha (\gamma +\beta )}}{R_{E}}$$

Mass transfer control phase: the electrocatalytic oxidation process of organic matter on the surface of anode and particle electrode is in a state of mass transfer control. Calculate the *ACE* and *E_sp_* formulas according to the steps of the mixed control phase:

(17) $$\mathrm{ICE}={\left[\frac{\lambda (\gamma +\beta )}{\beta (1+\lambda )}\right]}^{\lambda }\cdot \exp\left[-(1+\lambda )\frac{k_{m}}{{\varepsilon x}_{0}}t+\frac{\left(1-\alpha \gamma )(1+\lambda \right)}{\alpha (\gamma +\beta )}\right]$$

(18) $$\tau =\left\{\frac{\left(1-\alpha \gamma \right)}{\alpha (\gamma +\beta )}-\frac{1}{\gamma +\lambda }\ln\left[{\left(\frac{\beta }{\lambda }\right)}^{\lambda }{\left(\frac{1+\gamma }{\gamma +\beta }\right)}^{\left(1+\lambda \right)}\frac{{(1-R}_{E})}{\alpha }\right]\right\}\cdot \frac{{\varepsilon x}_{0}}{k_{m}}$$

(19) $$\mathrm{ACE}=\frac{R_{E}}{1-\alpha \gamma \left\{1+\frac{\gamma +\beta }{\gamma (1+\gamma )}\ln\left[{\left(\frac{\beta }{\gamma }\right)}^{\lambda }{\left(\frac{1+\lambda }{\gamma +\beta }\right)}^{1+\lambda }\frac{{1-R}_{E}}{\alpha }\right]\right\}}$$

(20) $$E_{\mathrm{sp}}=\frac{\mathrm{FU}}{8\times 3600(\gamma +\beta )}\frac{1-\alpha \gamma \left\{1+\frac{\gamma +\beta }{\gamma (1+\gamma )}\ln\left[{\left(\frac{\beta }{\gamma }\right)}^{\lambda }{\left(\frac{1+\lambda }{\gamma +\beta }\right)}^{1+\lambda }\frac{{1-R}_{E}}{\alpha }\right]\right\}}{R_{E}}$$

It can be seen in the *F* distribution table that the *F*_0.05_ (5.5) = 5.05 and *F* < *F*_0.05_ (5.5) (Table 2). Thus, there is no significant difference between the experimental data and the calculated value of the model at the confidence interval *α* = 0.05. The phase-reaction kinetic model can fit the energy efficiency results in the MO oxidation process by using ACPER with a *β*-PbO_2_/Ti anode.

The *ACE* decreases, and the *E_sp_* increases gradually with the reaction time under three current density conditions (Figure 7). In the current control phase, though the applied reaction current is fully utilized, the MO cannot be completely oxidatively degraded. As the reaction proceeds, the system enters the current/diffusion mixed phase. The *ACE* is slightly reduced, and the *E_sp_* does not increase substantially. In the final reaction phase, the MO is completely degraded, but the *E_sp_* rapidly increases, which causes the uncontrollable costs.

It is clear that the present way to improve the degradation efficiency is to extend the reaction time or operating current density. This will increase the *E_sp_* uncontrollably. This is a difficult problem in the use of electrochemical oxidation technology, which limits its large-scale industrial application. During the entire MO degradation reaction process of ACPER, when there is a buffer period between the current control and the diffusion control process, the performance was enhanced. Thus, the reaction can be adjusted in the mixed-control phase to realize the high efficiency and low consumption target.

## 4. Conclusions

Herein, the performance evaluation, electrode characterization and model simulation of ACPER system for MO simulated wastewater were studied by using a *β*-PbO_2_/Ti electrode. The ACPER system is beneficial for the removal the COD value of MO solution. The *β*-PbO_2_/Ti anode has strong electrocatalytic activity, and the AC electrode can expend the electrode surface area by approximately 63%. Furthermore, the phase-reaction kinetic model was a suitable fit to the *ACE* and *E_sp_* results. This kinetic model is also applicable to other wastewaters. Researchers have used an ACPER to treat phenol solutions at different conditions, and it was shown that the calculated data using this kinetic model were in close agreement with experimental data [42]. Thus, these results enrich the application of the phase-reaction kinetic model in ACPER, and provide theoretical support for the wide application of this kinetic model.

## Figures and Tables

**Table 1:** The simulation results of the recorded EIS data in the absence and presence of AC at a bias potential of 1.80 V vs. SCE.

	*R_oe_* (Ω)	*R_ct_* (Ω)	*γ*	*β*
**with AC**	1.34·10^4^	0.05308	0.80	0.20
**without AC**	409.2	100	0.80	-

**Table 2:** *F*-test of *ACE*, *E_sp_* between the experimental results and model estimation during MO oxidation on *β*-PbO_2_/Ti.

*J* (mA·cm^−2^)	3.0	5.0	10.0
***ACE***	0.90	0.95	1.30
***E_sp_***	0.60	0.86	2.61

## Data Availability

Not applicable.

## Supplementary Materials

The following supporting information can be downloaded at: https://www.mdpi.com/article/10.3390/ijerph19084775/s1. Figure S1. The polarization curve of *β*-PbO_2_/Ti anode in 3% Na_2_SO_4_ solution. Polarization test scanning speed is 10 mV·s^−1^, potential range 0 V ~ 2.0 V vs. SCE; Figure S2. MO concentration-absorbance standard curve.
