# Supplementary material for: Electrochemical Oxidation of Methyl Orange in an Active Carbon Packed Electrode Reactor (ACPER): Degradation Performance and Kinetic Simulation
Source: Int J Environ Res Public Health. 2022 Apr 14;19(8):4775. doi: 10.3390/ijerph19084775 (PMC9028912; doi:10.3390/ijerph19084775)
Supplement: Supplementary file 1 [file ijerph-19-04775-s001.zip › ijerph-1648086-supplementary.pdf]

Electrochemical oxidation of methyl orange in an active carbon packed electrode reactor (ACPER): degradation performance and kinetic simulation

Supplemetary Information

1. Polarization test

The polarization curves of β-PbO_2/Ti anode without phenol are described in Figure S1. Seen from this figure, the oxygen evolution potential (OEP) values of the β-PbO_2/Ti anode is 1.70 V *vs.* SCE.

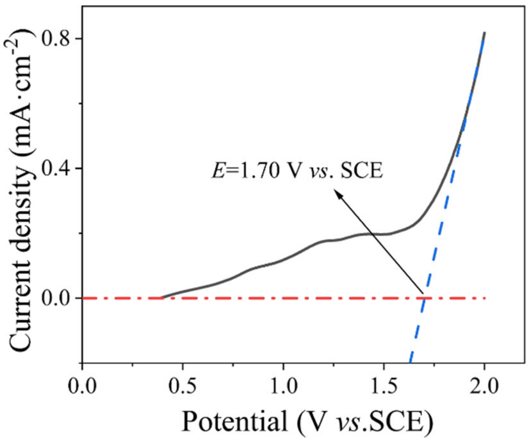

Figure S1. The polarization curve of β-PbO_2/Ti anode in 3% Na_2SO_4 solution. Polarization test scanning speed is 10 $mV{\cdot}s^{-1}$, potential range 0 V ~ 2.0 V *vs.* SCE.

2. Bulk MO simulated wastewater degradation experiment

2.1 ACPER working mechanism

The experimental setup was composed by eight quadrangular units made of polymethyl methacrylate plastics (Figure 1A). Each unit was equipped with two water reservoirs, a metering pump provided by Shanghai Chuding Corporation, China (model: BT-300), a direct current power supply purchased from Shenzhen Hopetech Company with model M8811 and a pair of electrodes. Prior to the experiments, GAC was washed by deionized water several times to remove the impurities and dried in an oven at 105 °C for 12 h to a constant weight. Then, the MO solution with direction perpendicular to that of current was directly pumped into the cell under a continuous flow mode and treated by single pass across the system at room temperature.

2.2 COD_t/COD_0 calculation method

The COD is tested by the standard dichromate methods (in APHA, Standard methods for the examination of water and wastewater, Washington, 1960). The formula for calculating COD_t/COD_0 is as follows:

$$COD_t / COD_0 = \frac{COD_t}{COD_0} \quad (1)$$

Where COD_t is the *COD* value at time *t*, mg·L^{-1}; COD_0 is the *COD* value at the initial moment, mg·L^{-1}.

3. Calculation method of MO decolorization rate

Methyl orange (MO) is a commonly used color developer in the laboratory. Within a certain concentration range, its concentration is proportional to the absorbance, and the absorbance can represent the chromaticity of MO. Therefore, UV-visible spectrophotometer was used to measure the absorbance of MO to express its concentration.

3.1 Standard curve

To prepare 100 mg·L^{-1} MO solution, add 0.00 mL, 0.50 mL, 1.00 mL, 2.00 mL, 2.50 mL, 5.00 mL to 50 mL colorimetric tubes, respectively. Add deionized water to dilute to the mark, shake well, and the solution concentration in the colorimetric tube is 0.00 mg·L^{-1}, 1.00 mg·L^{-1}, 2.00 mg·L^{-1}, 4.00 mg·L^{-1}, 5.00 mg·L^{-1}, 10.00 mg·L^{-1}, respectively. Using deionized water as a control, the absorbance value of the solution was measured at a wavelength of 464 nm, and the curve of absorbance and MO concentration was drawn. Figure S2 is the obtained standard curve, and its fitting equation is formula (1).

$$\begin{aligned} y &= 0.0676x + 0.0004 \\ R^2 &= 0.9999 \end{aligned} \quad (2)$$

Where *x* is the absorbance; *y* is the concentration of MO, mg·L^{-1}. The fitted correlation coefficient is greater than 0.998.

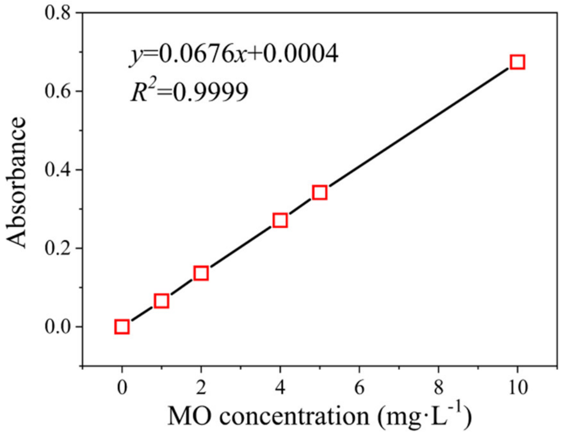

Figure S2. MO concentration-absorbance standard curve.

3.2 Determination of MO concentration

After sampling the effluent of each stage, transfer 5mL to a 50mL colorimetric tube. The measurement method is the same as that of the MO standard curve measurement. The MO concentration can be calculated according to the fitting equation of the standard curve.

3.3 Calculation of MO decolorization rate(η)

The formula for calculating the decolorization rate is formula (2).

$$\eta = \frac{C_{in-MO} - C_{out-MO}}{C_{in-MO}} \times 100\% \quad (3)$$

where C_{in-MO} is the MO influent concentration, mg·L^{-1}; C_{out-MO} is the MO effluent concentration, mg·L^{-1}.
